# Supplementary material for: Effect of Dietary Brown Seaweed (Macrocystis pyrifera) Additive on Meat Quality and Nutrient Composition of Fattening Pigs
Source: Foods. 2021 Jul 26;10(8):1720. doi: 10.3390/foods10081720 (PMC8393841; doi:10.3390/foods10081720)
Supplement: Supplementary file 1 [file foods-10-01720-s001.zip › foods-1228295-SI.pdf]

Suplementary Table S1. Descriptive statistics for fatty acids composition (*) and health index in pork *longissimus lumborum* muscle.

Variable	Mean	SD	Minimum	Maximum
C14:0	1.37	0.14	1.04	1.54
C16:0	23.80	1.09	22.45	26.44
C18:0	13.23	1.49	10.66	16.04
C20:0	0.003	0.006	0.001	0.003
C22:0	0.06	0.02	0.03	0.1
∑ SFA[1]	39.96	2.39	36.11	44.19
C14:1	0.012	0.004	0.01	0.02
C16:1	2.51	0.86	0.03	3.98
C17:1	0.19	0.05	0.09	0.30
C18:1*c* ω9	43.93	2.09	40.65	48.17
C20:1*c* ω9	0.04	0.02	0.01	0.07
∑ MUFA	48.80	2.46	45.51	53.21
C18:3 ω3	0.47	0.11	0.33	0.64
C20:5 ω3	1.24	0.40	0.67	2.15
C18:2 ω6	2.11	1.51	6.11	10.99
C20:4 ω6	0.66	0.07	0.57	0.77
C18:3 ω3	0.47	0.11	0.33	0.64
∑ ω 3	1.71	0.38	1.09	2.55
∑ ω 6	0.65	0.07	0.51	0.77
∑ PUFA	10.76	1.77	8.39	13.70
Total trans	1.99	0.55	0.68	2.97
H/h	0.46	0.037	0.40	0.54
IA	0.56	0.04	0.49	0.68
IT	0.18	0.14	0.98	1.52
MUFA/SFA	1.22	0.12	1.03	1.46
PUFA/SFA	0.27	0.05	0.9	0.35
ω6/ω 3	0.41	0.11	0.27	0.62

*:values are expressed as g/100 g of fresh muscular tissue. SD: Standard Deviation. CV: Coef [illegible]
Sum of Saturated Fatty Acids. [2]:Sum of Monounsaturated Fatty Acids. [3]:Sum of Polyunsaturat[illegible]
H/h=hypercholesterolemic/hypocholesterolemic index: C14:0+C16:0)/ (C18:1+C18:2+C18:3[illegible]
C20:5+C22:4+C22:5+C22:6). IA = Atherogenic index: (C12:0 + 4 × C14:0 + C16:0 /[ΣAGM[illegible]
IT = Thrombogenic index: (C14:0 + C16:0 + C18:0)/[0,5 × ΣAGMI + 0,5 × Σ(ω-6) + 3 × Σ(ω[illegible]

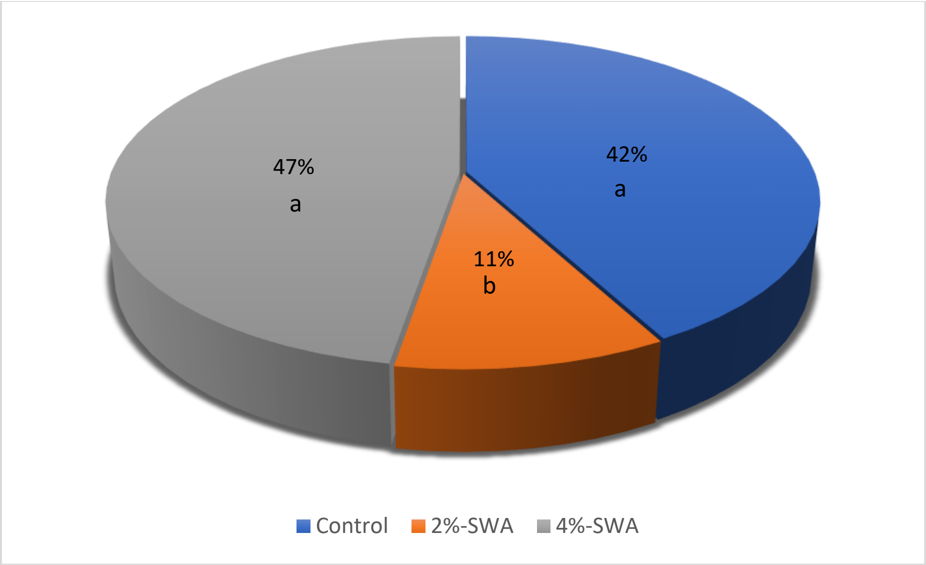

Figure S1. Taste preference percentage by panelists. χ^2 test. Different letters indicate significant different ($P < 0.05$) among group´s frequencies.
